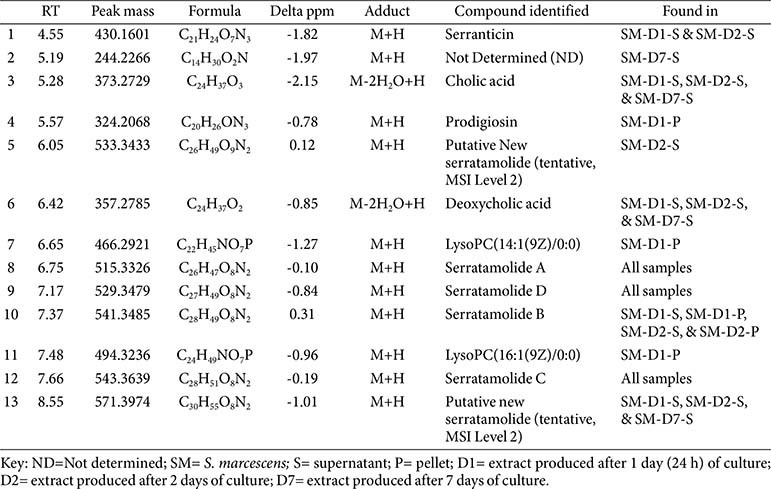# Chemical Profiling and Antibacterial, Anti-Biofilm, and Antioxidant Activities of Endophytic *Serratia marcescens* AI-N-1 from *Azadirachta indica*

**DOI:** 10.4014/jmb.2508.08044

**Published:** 2025-11-26

**Authors:** Hissein Hassan Moussa, Mubo Adeola Sonibare, Jin-Soo Park

**Affiliations:** 1 Pan African University Life and Earth Sciences Institute (including Health and Agriculture), Ibadan, Oyo State, Nigeria; 2 Center for Natural Product Systems Biology, KIST Gangneung Institute of Natural Products, Gangneung 25451, Republic of Korea; 3 Biodiversity Conservation and Medicinal Plants Research Group, Department of Pharmacognosy, Faculty of Pharmacy, University of Ibadan, Ibadan, Nigeria; 4 Directorate, Pan African University Life and Earth Sciences Institute (including Health and Agriculture), Ibadan, Oyo State, Nigeria; 5 Natural Product Applied Science, KIST School, University of Science and Technology, Gangneung 25451, Republic of Korea

**Keywords:** Bacterial endophyte, *Serratia marcescens*, bioactive compounds, MS/MS based chemical profiling

## Abstract

The rising need for new antibiotics and antioxidants highlights endophytic bacteria as promising sources of bioactive compounds. Medicinal plants such as *Azadirachta indica* harbour diverse endophytes, yet their potential in southwest Nigeria remains largely underexplored. This study investigated the antimicrobial, biofilm inhibitory, and antioxidant activities of bioactive compounds produced by the bacterial endophyte *Serratia marcescens* AI-N-1, isolated from *A. indica*. Crude extracts of *S. marcescens* showed strong antimicrobial activity against *Bacillus subtilis* (79.79% inhibition) and *Salmonella typhi* (77.04% inhibition) at 5 mg/ml. In addition, most extracts also displayed potent biofilm inhibition (>80%) against both pathogens, comparable to the positive control baicalein (*P* < 0.05). Antioxidant assays revealed high radical scavenging activity, with the supernatant extract obtained after 2 days of culture exhibiting the strongest effect (DPPH: 86.61% at 0.1 mg/ml; ABTS: 99.64% at 0.1 mg/ml). Online HPLC–ABTS^+^ analysis identified serranticin as a major contributor to these antioxidant effects. HR-MS/MS profiling further revealed prodigiosin, serratamolides, and serranticin, along with putative novel lipopeptides and other metabolites, as key bioactive compounds. To our knowledge, this is the first report of a Serratia endophyte from *A. indica* in southwest Nigeria with combined antimicrobial, antibiofilm, and antioxidant activities, as well as the discovery of putative new lipopeptides. These findings highlight endophytic bacteria from Nigerian medicinal plants as promising sources of novel antimicrobial and antioxidant agents for pharmaceutical development.

## Introduction

*Azadirachta indica*, commonly known as the neem tree, is native to the Indian subcontinent and is widely distributed in tropical and subtropical regions across Asia, Africa, and the Americas [1]. The plant has held a privileged position in traditional medicine in southwest Nigeria, used to treat various diseases. The decoction of leaves is used for the management of malaria, fever, digestive ailments, intestinal worms, and diabetes [2]. Different parts of the neem tree, including the leaf, stem, bark, and root, play distinct roles in disease management through the modulation of various biological activities. The active compounds of *A. indica* include azadirachtin, nimbidin, nimbin, nimbidol, gedunin, salannin and meliatriol. These compounds are reported to be responsible for the medicinal properties of *A. indica* [1]. These compounds have shown notable, among others, antibacterial, anti-inflammatory, antioxidant, antipyretic, hypoglycemic, insecticidal, and anticancer activities [3]. The global research effort for novel antibiotics and antioxidant molecules has become necessary. This need arises from the growing prevalence of drug resistance and oxidative stress–induced disorders worldwide [4]. Bacterial biofilm formation and quorum-sensing phenomena are closely associated with the mechanisms of bacterial pathogenicity and resistance [5]. Thus, biofilms have become an important molecular target in drug research against bacterial resistance. Endophytes are harmless microbes that live within the tissues of healthy plants. These plants harbour many endophytic bacterial and fungal species from different genera, which provide access to a diverse array of metabolically important chemicals and act as a sustained reservoir of natural products [6, 7]. Previous reports showed the possibility of secondary metabolites produced by endophytes as a new origin of bioactive substances for drug development [8]. The report on endophytic microbial flora from African medicinal plants implied that more secondary metabolites could be explored, especially from Nigerian medicinal plants [9]. This could hence be indicative that endophytic microbial diversity present in the medicinal plants of southwest Nigeria may offer possibilities for complex secondary metabolites as effective molecules from natural products [10]. Nevertheless, there is insufficient extensive research on the bioactivities derived from secondary metabolites of bacterial endophytes present in local medicinal plants in this particular region of Nigeria. This study aimed to investigate the antibacterial, biofilm inhibitory, antioxidant activities and metabolite profiling of bioactive compounds obtained from a bacterial endophyte isolated from *A. indica* native to southwest Nigeria.

## Materials and Methods

### Plant Collection and Identification

Samples of stem-barks from *A. indica* A. Juss. (Meliaceae) were collected from Ibadan, Oyo State, southwest Nigeria (721'2.48" N 351'55.84" E). The plant was identified by Mr. Donatus Esimekhuai at the University of Ibadan Herbarium (UIH, Nigeria), where a voucher specimen was deposited for future reference under UIH-23424.

### Sample Preparation and Isolation of Bacterial Culture

Sample preparation procedures were carried out as previously reported by Ezeobiora *et al*. [7] and Tsuchida *et al*. [11]. Plant stem-bark samples were surface-sterilised by sequential washing in 90% ethanol (5 min), distilled water, sodium hypochlorite (3 min), distilled water, 70% ethanol (1 min), and rinsed three times with distilled water. Complete sterility of plant samples was confirmed by plating the final water rinse on nutrient agar (NA), and this was incubated at 30°C for 48 h. Inner tissues were aseptically excised and placed on NA supplemented with nystatin (30–50 μg/ml). Plates were incubated at 30°C for 48 h to 2 weeks. Morphologically distinct colonies were subcultured onto fresh media and purified. Pure isolates were preserved in 50 % glycerol at −80°C for long-term storage.

### Molecular Characterisation of Bacterial Endophytes

The molecular characterisation of bacterial endophyte isolates was carried out as reported by Ezeobiora *et al*. and Wright *et al*. [7-12]. Bacterial isolates were characterised by sequencing the 16S Region. Polymerase chain reaction (PCR) and DNA sequencing were employed for the identification of new endophytes. The 16S rRNA sequencing was performed to identify bacteria using universal primers (27F and 1492R). The amplified DNA was sequenced and compared to a database, and this was used to determine the bacterial endophytes [12].

**Preparation of endophytic isolates for DNA extraction.** Based on the morphological and observational characteristics of the endophytic isolates seen visually, the endophytic cultures were purified after subculturing by being moved to some freshly prepared media plates (nutrient agar). Thus, the isolates were moved from the subcultured plates into various test tubes containing 5 ml of broth media after autoclaving (nutrient broth and MRS broth). The various liquid media with the isolates in the test tubes were cultured overnight using a shaking incubator at 160 rpm and 30°C. Following overnight incubation, 1 ml of each of the harvested isolate cultures was centrifuged at a speed of 15,000 rcf. The supernatants were discarded, and the cell pellets were left in the 1.5 ml microtubes. Therefore, these were ready to be employed for the genomic extraction of the different endophytic isolates.

**Extraction of genomic DNA and PCR amplification.** Genomic DNA was isolated from pure cultures of bacterial endophytes using the SolGent Genomic DNA Prep Kit (SolGent, Republic of Korea) in accordance with the manufacturer's guidelines. The bacterial cell pellets were treated with R1 buffer (300 μl) and lysozyme (2 μl), incubated at 37°C for 60 min (160 rpm), and then centrifuged (10,000 rcf, 1 min). The pellet was further treated with SG1 buffer (350 μl) and proteinase K (5 μl) at 65°C for 10 min and then combined with SG2 buffer (400 μl). Following centrifugation (10,000 rcf, 5 min), supernatants were transferred to spin columns, washed with 80%ethanol, and then eluted with 50 μl of DNA hydration solution. DNA purity (260/280 nm ratio: 1.8–2.0) and integrity (1% agarose gel) were validated. The 16S rRNA gene was amplified using primers 27F and 1492R in a 20 μl reaction (Taq PCR Premix: 1 μl template DNA, 1 μl of each primer, 17 μl of nuclease-free water). The established PCR conditions were: 95°C (5 min); 30 cycles of 95°C (20 sec), 55°C (40 sec), 72°C (90 sec); concluding with a final extension at 72°C (7 min). Amplicons (~1.5 kb) were visualised on a 1% agarose gel with Midori Green and then purified with the QIAquick Kit (Qiagen, Germany) using PB/PE buffers, followed by elution in EB buffer (30 μl). The eluted PCR products were sent for sequencing.

**Sequencing and sequence analysis.** The 16S rRNA gene of *S. marcescens* AI-N-1 was sequenced using Sanger sequencing (Macrogen, Inc., Republic of Korea). The findings of Sanger sequencing were analysed using BLAST (NCBI) based on query coverage, percent identity, E-value, and bit score. The identification of *S. marcescens* was determined using the closest NCBI match [13]. The 16S rRNA gene sequence of *S. marcescens* strain AI-N-1 has been deposited in the GenBank database under the accession number PX061904.

Phylogenetic placement of the bacterial endophyte *S. marcescens* isolated from *A. indica* was examined based on the 16S rRNA gene sequence as previously reported by Tamura *et al*. [14]. The sequence was aligned with seven closest Serratia reference sequences retrieved from the NCBI database. Sequences with 97–100% similarity were aligned via Multiple sequence alignment using the MUSCLE algorithm in MEGA11. The Tamura-Nei substitution model was used for the Maximum Likelihood tree. The initial tree(s) for heuristic search were generated automatically using the Neighbour-Join and BioNJ algorithms. Bootstrap analysis with 1000 replicates was conducted to assess the reliability of the inferred clades, and only values above 50% were included in the final tree. Eight sequences were used with a total alignment length of 1544 nucleotide positions.

### Production and Extraction of Endophytic Bacterial Culture

The identified bacterial endophyte isolates were streaked from frozen stocks on nutrient agar and incubated at 37°C for 24 h. Among all the isolates, only *S. marcescens* was investigated due to its relevance in producing potent metabolites [15, 16]. The single colonies observed from overnight agar plates were inoculated onto 5 ml of nutrient broth in test tubes, which were subsequently incubated overnight at 37°C in a shaking incubator set at 160 rpm. The 5 ml nutrient broth was divided into two parts, which were inoculated into sterile broth media in 100 ml flasks. For the initial screening of biological activities, small-scale cultures were prepared in a total volume of 80 ml using two media (nutrient broth and tryptic soy broth). These were shaken for 7 days at 160 rpm and 30°C. The secondary metabolites were directly extracted with ethyl acetate at the end of the cultivation process without separation. The extracts were concentrated under vacuum at 40°C using a rotary evaporator [17]. The endophytic crude extracts obtained were then evaluated for preliminary bioactivity.

Following the initial screening of biological activities, the bacterial endophyte *S. marcescens* was considered for large-scale cultures. These were prepared in a total volume of 2 L each. To achieve this, *S. marcescens* was cultured overnight on nutrient broth media (80 ml). These were transferred into 2.8 L Pyrex flasks. To facilitate optimal metabolite growth, each Pyrex flask contained only 1 L of culture. This operation was implemented to ensure that the bacteria received enough oxygen and to facilitate proper agitation of the cultures on the shaking incubator. These were cultivated for 7 days at 160 rpm and 30°C. During metabolite synthesis, a colour shift was observed in the broth cultures containing *S. marcescens* day after day. The daily variation in culture colouration suggested that the endophytic bacterium *S. marcescens* produced several secondary metabolites, leading to the designations D7, D2, and D1. *S. marcescens*-D7 indicated that the extract was generated after seven days of cultivation. D2 indicated that the extract was generated after two days of cultivation. D1 indicated that the extract was generated after 24 h of culture. The collected cultures of the endophytic bacterium *S. marcescens* were then centrifuged to separate the supernatants and cell pellets, which were then extracted using an organic solvent (1:1 v/v) of ethyl acetate and acetone, respectively. The solvents were removed from the crude extracts via a rotating vacuum evaporator. The endophytic crude extracts were concentrated using a speed vacuum concentrator, and the dried extracts were weighed using a digital scale. Ultimately, crude extracts were stored in sealed containers and kept in a refrigerator at -20°C for further use. Bacterial crude endophyte extracts were ready for repeated bioactivity evaluation and identification of bioactive metabolites.

### Antibacterial Susceptibility Test

The Broth micro-dilution technique was used to assess the antibacterial activity of the bacterial endophyte extracts. Bacterial pathogens (*B. subtilis* ATCC 6633 and *S. typhi* ATCC 14028) were streaked from frozen stock on Luria Bertani (LB) agar and cultured at 37°C for 24 h. The single colonies observed from overnight agar plates were inoculated onto 10 ml LB broth in 50 ml conical tubes. These were subsequently incubated overnight at 37°C in a shaking incubator set at 160 rpm. Then, using a spectrophotometer (SpectraMax ABS Plus, USA), the inoculum was standardised to OD_600_ = 0.1 turbidity standard. Using a multichannel pipette, 100 μl of the adjusted cell suspensions were distributed into each of the wells of a 96 plate. To standardise the procedure, directly after loading of the inoculum, 100 μl of test extracts, positive control or negative control were loaded into each well corresponding to the designated position in the 96 plates. The final well volume was 200 μl. Test extracts were dissolved in ethyl acetate or acetone, and the final solvent concentration in wells did not exceed 1% (v/v). By dissolving it in distilled water, Tetracycline was used as a positive control at 250 μg/ml. Luria Bertani broth, which was used as a medium to culture the bacterial pathogens, was considered a negative control. The 96-well plates underwent overnight incubation (18 h) at 37°C. The quantitative assessment was accomplished by determining the absorbance at 600 nm using a microtiter plate spectrophotometer reader (SpectraMax ABS Plus). If the microorganisms are susceptible to the extracts, the Optical Density 600 (OD_600_) of the samples should be smaller than the OD_600_ of the negative control (LB broth). The experiment involving the endophytic extracts was carried out in triplicate, and the results are presented as the means ± SD.

$$\text{The antibacterial percentage inhibition was determined as follows =}\frac{\text{Control OD}-\text{Sample OD}}{\text{Control OD}}\times 100$$

### Biofilm Inhibition Using Microtiter Plate Assays

The Crystal Violet approach was employed to determine the biofilm-inhibitory activity of the bacterial endophyte extracts as previously reported by Haney *et al*. [18]. Single colonies of the bacterial pathogens (*B. subtilis* ATCC 6633 and *S. typhi* ATCC 14028) were inoculated into 10 ml of LB broth in 50 ml conical tubes and cultured overnight at 37°C. Using a spectrophotometer (SpectraMax ABS Plus, USA), the inoculum was standardised to OD600 = 0.01 turbidity standard. The experiment was divided into 3 important steps: plating, staining and quantification.

The plating started with the distribution of 200 μl of the adjusted cell suspensions into the wells of a 96-well plate via a multichannel pipette. To carry out the method uniformly, directly after loading of the inoculum, 100 μl of test extracts, positive control or negative control were introduced into each well corresponding to the assigned location in the 96 plates. The negative control wells contained LB liquid medium and the corresponding pathogenic cultures without the test extracts, while baicalein was included as a positive control at 128 μg/ml. The final well volume was 300 μl. Test extracts were dissolved in ethyl acetate or acetone, and the final solvent concentration in wells did not exceed 1% (v/v). To facilitate cell adhesion and biofilm development, the plates were placed in a moist chamber, which consisted of a plastic box with dampened tissue and incubated at 37°C for 48 h.

Following incubation, the crystal violet test was conducted, where the fluids of all wells were thoroughly removed. This second step is called staining. The microtiter plates were carefully cleaned three times with sterile distilled water to remove weakly adherent cells. Following washing, the wells were stained with 250 μl of 4%Hucker's crystal violet and left to stand at ambient temperature for 10 minutes. After that, the wells were cleaned three times with de-ionised water and air-dried for 60 min.

Following drying, the quantification step took place, where 200 μl of 30% glacial acetic acid was applied to dissolve the colour-bound to the attached cells. The dissolved biofilms were transferred to new plates for accurate measurement. The plates were then read using a microplate reader (SpectraMax ABS Plus, USA) at an OD of 595 nm. The experiments involving the endophytic extracts and the fermented extracts were conducted in triplicate, and the results are presented as the means ± SD. The OD_595_ values of the treated cells, which were the wells with test extracts, were compared with those of the untreated cells, which were the LB broth wells, to assess the increase or decrease in attachment because of extract administration.

$$\text{The biofilm percentage inhibition was determined as follows}=\frac{\text{Control OD}-\text{Sample OD}}{\text{Control OD}}\times 100$$

### DPPH Scavenging Activity: 2, 2-Diphenyl-1-Picrylhydrazyl Assay

The 2,2-diphenyl-1-picrylhydrazyl (DPPH) assay was conducted following the technique reported by Sonibare *et al*. [19] with some changes. First, a stock solution of 20 mM DPPH was made in methanol. This mixture was diluted to attain a concentration of 0.1 mM. In the next step, 90 μl of the newly made DPPH solution (0.1 mM) was added to each well of the 96-well plate containing 10 μl of the test samples at two different concentrations (1 mg/ml and 0.5 mg/ml). The same was carried out for the positive control (1 mM Ascorbic acid~0.176 mg/ml), negative control (methanol) and blank (distilled water). After homogenisation using a multichannel pipette, the mixtures were incubated under ambient conditions in the dark for 10 min. Following incubation, at 515 nm, the OD was measured using a microplate reader (SpectraMax ABSPlus, USA). The inhibition rate of the DPPH free radical was calculated using the corresponding formula:

$$\text{DPPH Scavenging activity \%}=\frac{\text{Absorbance of control-Absorbance of sample}}{\text{Absorbance of control}}\times 100$$

### ABTS Scavenging Activity: 2,2-Azinobis (3-Ethylbenzothiazoline-6-Sulfonic Acid) Assay

The ABTS test was conducted similarly to the technique reported by Thaipong *et al*. [20] with minor adjustments. The stock solution of ABTS was prepared with the aim of generating ABTS+ radical cations. To achieve this, equal amounts of ABTS (14 mM) and potassium persulfate (4.9 mM) were combined in distilled water. The resulting solution was allowed to homogenise in the shade for 24 h at room temperature. At the end of incubation, ABTS+ was formed. The dark blue ABTS+ reagent mixture was adjusted with distilled water until the absorbance reached 0.70 ± 0.02 at a wavelength of 734 nm.

In the subsequent stage, a 96-microwell plate was employed, wherein 90 μl of a newly equilibrated ABTS solution was combined with 10 μl of test extracts at two different concentrations (1 mg/ml and 0.5 mg/ml). The same process was performed on the positive control (1 mM Ascorbic acid~0.176 mg/ml), negative control (distilled water), and blank (methanol). Following homogenisation using a multichannel pipette, the mixtures were incubated under ambient conditions in the shade for 10 minutes. Following incubation, at 734 nm, the OD was measured using a microplate reader (SpectraMax ABSPlus). The inhibition rate of the ABTS free radical was calculated using the corresponding formula:

$$\text{ABTS Scavenging activity \% =}\frac{\text{Absorbance of control-Absorbance of sample}}{\text{Absorbance of control}}\times 100$$

### Online High-Performance Liquid Chromatography (HPLC)–ABTS+ Based Assay

To identify antioxidant constituents present in the endophytic bacterial extracts, an online HPLC-ABTS+ assay was performed following the procedure described by Abdel-razakh *et al*. [21]. The analytical setup consisted of an Agilent 1200 series HPLC system (Agilent Technologies, USA) equipped with an additional pump to supply the ABTS radical solution. A stock solution of ABTS was prepared by dissolving 2 mM ABTS and 3.5 mM potassium persulfate in distilled water. This solution was diluted eightfold using HPLC-grade water to generate the working ABTS radical reagent. To allow radical formation and stabilisation, the solution was kept in the dark at room temperature for approximately 12–16 h before use. For each run, 10 μl of the endophytic extract was injected into the system. Separation of individual antioxidant compounds was achieved using a Phenomenex Luna C18(2) column (4.6 × 150 mm, 5 μm particle size) (Phenomenex, USA), with a mobile phase consisting of solvent A (0.1%formic acid in acetonitrile) and solvent B (0.1% formic acid in water). The elution was carried out under gradient conditions as follows: 0–10 min, 10% A; 10–30 min, a linear increase to 100% A; followed by a return to the initial conditions. The flow rate was set at 1.0 ml/min, and the column temperature was constant at 25°C throughout the analysis. The ABTS radical solution was introduced into the system at a constant flow of 0.7 ml/min. Chromatographic data were captured as positive peaks, while antioxidant activity was detected based on the reduction in ABTS radical absorbance, monitored as negative peaks using a visible detector set at 734 nm. Data acquisition and analysis were carried out using ChemStation software version B.04.03 (Agilent Technologies, USA).

### High-Resolution Mass Spectrometry with Tandem Mass Spectrometry Analysis (HR-MS/MS)

To identify the various types of bioactive metabolites, the endophytic extracts were subjected to spectroscopic analyses, providing high-accuracy data for metabolite identification. With the aid of previously defined standard procedures reported by Park *et al*. [22], High-resolution MS/MS (HR-MS/MS) analysis was carried out on bacterial endophyte extracts to identify the bioactive compounds produced. HR-MS/MS spectra of the endophytic extracts were generated using a Q Exactive Quadrupole-Orbitrap mass spectrometer (Thermo Fisher Scientific, USA) with an ACQUITY UPLC BEH 1.7 μm C18 130 Å column (2.1 × 100 mm) (Waters, USA). The LC-MS/MS data collected from the bacterial endophyte extracts were then transformed into mzXML format, which is compliant with the Global Natural Products Social Molecular Networking (GNPS) platform, using MSConvert (v3.0.11244). Once the raw data had been transferred to the GNPS server using the WinSCP FTP client, the molecular networks were produced on the GNPS analysis platform, incorporating a filtration stage. This involved the elimination of all MS/MS fragment ions within a ± 17 Da range of the precursor m/z measurement. The tolerance of the precursor ion mass was fixed at 2.0 Da, and the tolerance of the MS/MS fragment ion was fixed at 0.5 Da. The relatively wide precursor (2.0 Da) and fragment (0.5 Da) tolerances were selected to maximise metabolite coverage. Notably, key nodes and major metabolites remained reproducible under stricter tolerances, ensuring annotation reliability. To obtain a visual representation of these networks, Cytoscape was employed (v3.8.2). This facilitated the investigation and analysis of the interconnected chemical networks among the bioactive secondary metabolites of the bacterial endophyte extracts, providing a comprehensive overview of their chemical diversity and metabolite identities (Fig. S2).

### Data Analysis

In this study, all assays were performed in triplicate (*n* = 3), and data are expressed as mean ± standard deviation (SD). Statistical analyses were conducted using SPSS software (version 27.0; SPSS Inc., USA). One-way analysis of variance (ANOVA) was applied to evaluate differences among treatment groups, followed by Duncan’s multiple range test for post hoc comparisons at a significance level of *p* ≤ 0.05. GraphPad Prism software (version 10.231; GraphPad Software, USA) was used to generate graphical representations of the DPPH and ABTS scavenging activities of the bacterial endophyte extracts.

### Data Availability

The GenBank/EMBL/DDBJ accession number for the 16S rRNA gene sequence of *Serratia marcescens* strain AI-N-1 reported in this paper is PX061904.

## Results and Discussion

The present study explores the antimicrobial, biofilm inhibitory, and antioxidant activities of bioactive compounds produced by the bacterial endophyte *S. marcescens* AI-N-1. The strain was isolated from *A. indica* collected in southwest Nigeria, a medicinal plant known to host endophytes capable of producing metabolites with diverse biological activities.

**Fig. 1:**
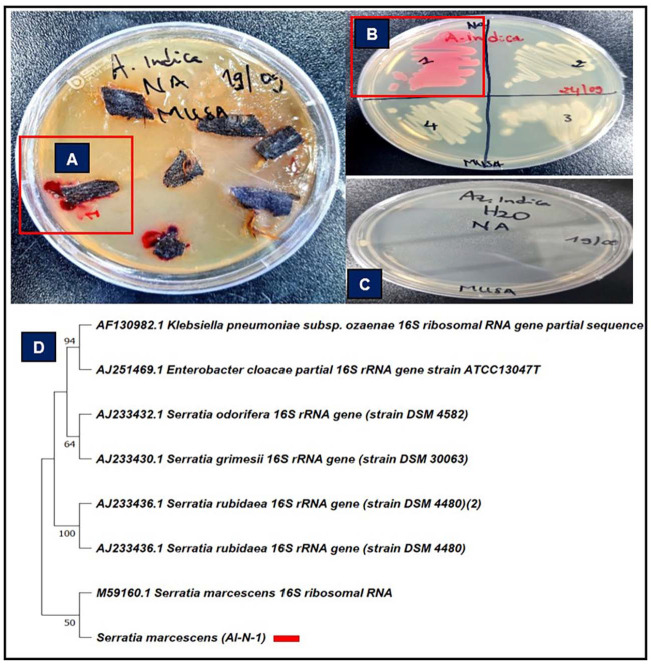
Isolation of *S. marcescens* from *A. indica*. (**A**) Plating of *A. indica*’s stem-barks to isolate *S. marcescens* using nutrient agar. (**B**) Subculturing of *S. marcescens* with code number ‘‘1’’ showing red colour pigment. (**C**) Plating of aliquots from the final rinse water used in surface sterilisation showing no microbial growth. (**D**) Phylogenetic tree based on 16S rRNA gene sequences of *S. marcescens* isolate and closely related strains. The tree was constructed using the Maximum Likelihood method with the Tamura-Nei model in MEGA11. Bootstrap values (>50%) from 1000 replicates are shown at the nodes. The analysis included 8 sequences with a total alignment length of 1,544 bp.

Emphasis was placed on the isolate *S. marcescens*, designated by the code AI-N-1, as the molecular characterisation of isolates through gene-based analysis revealed variability in the endophytic bacteria strains isolated from *A. indica* (Fig. 1). The phylogenetic analysis based on the 16S rRNA gene sequence revealed that the *S. marcescens* AI-N-1 clustered closely with other *S. marcescens* strains, confirming its taxonomic identity within the genus. The isolate shared a high sequence similarity and evolutionary proximity with its closest relatives, indicating a conserved genetic lineage.

*S. marcescens* is a multifaceted bacterium proficient in synthesising many bioactive chemicals with antimicrobial characteristics. Studies have identified numerous secondary metabolites in *S. marcescens*, such as prodigiosin, oleic acid, serrawettin homologues, etc. [16, 23].

The production of secondary metabolites from *S. marcescens* was initiated, and the extracts obtained were used to perform biological assessments, including antibacterial assay, biofilm inhibition assay, and antioxidant assays, as well as metabolite profiling.

### Antibacterial Susceptibility Test Results

Regarding the antibacterial evaluation summarised in Table 1, two concentrations (5 mg/ml and 1 mg/ml) were used to test the endophytic extracts against two bacterial pathogens: *B. subtilis* ATCC 6633 and *S. typhi* ATCC 14028. The preliminary results showed that all the endophytic extracts exhibited antimicrobial activity against both pathogens. The extract that showed the most impressive and dominant antimicrobial activity was *S. marcescens*-NB. At 5 mg/ml, it demonstrated 79.79% inhibition against *B. subtilis* and 77.04% inhibition against *S. typhi*. At 1 mg/ml, the *S. marcescens*-NB extract displayed 61.68% inhibition against *B. subtilis* and 58.35%inhibition against *S. typhi*. These results are in line with previous reports supporting the antimicrobial potential of *S. marcescens*. For instance, Idris and Adetunji reported that extracts obtained from the endophytic bacterium *S. marcescens* isolated from *Bryophyllum pinnatum*, a medicinal plant native to Madagascar, showed zones of inhibition of 17.7 and 12.7 mm against *Klebsiella pneumoniae* and *Staphylococcus sciuri*, respectively [24]. In another study by Clement *et al*., the crude extract produced from *S. marcescens* presented the highest broad-spectrum antimicrobial activity. The pigmented *S. marcescens* strain named P1 showed the highest activity, accounting for 63.6% of the Gram-negative bacteria and 90% of the Gram-positive bacteria. The highest activity against Gram-negative bacteria included *Pseudomonas aeruginosa* with an inhibition zone of 18 mm. The highest activity against Gram-positive-positive bacteria included *Listeria monocytogenes* with a zone of inhibition of 24.7 mm [23]. These findings indicate that *S. marcescens* has promising antimicrobial capabilities. It appears that *S. marcescens* is well conserved among diverse environments and host systems, including in the plants of southwest Nigeria. Variations in activities may thus be due to environmental factors [25].

### Biofilm Inhibition Assay Results

In the biofilm inhibition experiments shown in Table 1, the majority of extracts exhibited strong biofilm inhibition exceeding 80% against both pathogens at 5 mg/ml. Notably, their efficacy against *S. typhi* was statistically comparable (*p* < 0.05) to that of the reference compound baicalein (128 μg/ml), which showed 81.97% inhibition. *S. marcescens*-NB and *S. marcescens*-TSB inhibited *S. typhi* by 82.23% and 81.86%, respectively (*p* = 0.001). Against *B. subtilis*, *S. marcescens*-NB and *S. marcescens*-TSB achieved 61.11% and 81.47% inhibition, respectively (*p* = 0.001). These results are consistent with the findings of Hazarika *et al*., who reported >90% inhibition of early-stage biofilms in *B. subtilis* and *B. cereus* by prodigiosin at 50 μg/ml, a pigment biosynthesised by *S. marcescens*. Moreover, prodigiosin was capable of disrupting established biofilms, reducing crystal violet retention by up to 74% at 100 μg/ml [26]. The discrepancy in effective concentrations between our crude extracts and the purified prodigiosin used by Hazarika *et al*. may be attributed to the presence of a complex mixture of metabolites in the extracts and may reflect synergistic effects of multiple metabolites, which likely include, but are not limited to, prodigiosin. That is why the establishment of their metabolite profiling was necessary. Additionally, the mechanism of biofilm inhibition by *S. marcescens* metabolites may involve oxidative stress-mediated nucleic acid damage, as described by Kimyon *et al*. Their study provided a mechanistic perspective by linking the antibiofilm potential of prodigiosin produced by *S. marcescens* to its nucleic acid-cleaving activity. Their study illustrated the biofilm-inhibiting activity of prodigiosin against *P. aeruginosa* through oxidative damage to nucleic acid molecules, suggesting that redox-active metabolites could be present in our extracts, and these may contribute to similar effects [27].

The strong biofilm inhibition activity shown by *S. marcescens* AI-N-1 is particularly relevant in the context of antibiotic resistance. In pathogenic bacteria, biofilms act as protective barriers that limit antibiotic penetration and promote tolerance, thereby contributing to chronic and recurrent infections. By disrupting biofilm formation, metabolites from *S. marcescens* may enhance the efficacy of conventional antibiotics, resensitize resistant pathogens, and provide an adjunct strategy for managing persistent infections associated with multidrug resistance.

### Antioxidant Scavenging Activities

To fully investigate the metabolites produced by the bacterial endophyte *S. marcescens*, it was important to test their antioxidant properties. The antioxidant tests carried out were the ABTS and the DPPH assays.

**Fig. 2:**
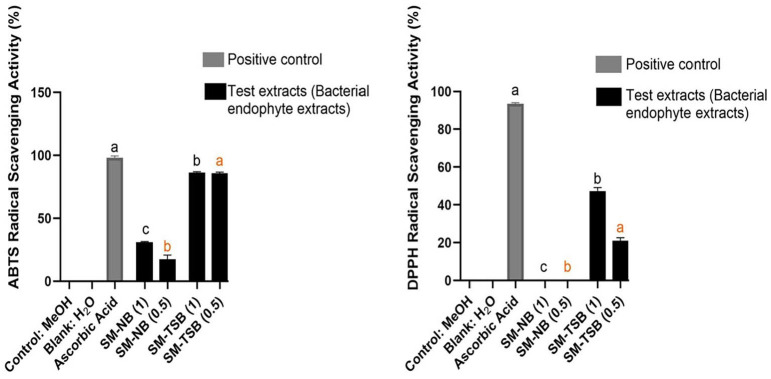
DPPH and ABTS radical scavenging activities of *S. marcescens* extracts Key: SM= *S. marcescens*; NB= nutrient broth; TSB= Tryptic soy broth; (1) = final concentration of 0.1 mg/ml; (0.5) = final concentration of 0.05 mg/ml. Different superscripts (^a–c^) indicate significant differences based on Duncan’s test at *P* ≤ 0.05. Superscript colors indicate separate statistical analyses: black (0.1 mg/ml) and orange (0.05 mg/ml). Units are expressed consistently as mg/ml. Positive control (1 mM ascorbic acid, ~0.176 mg/ml) is presented for comparison.

In the ABTS assay shown in Fig. 2, the *S. marcescens*-TSB extract demonstrated important activity at both concentrations. It displayed an ABTS scavenging activity of 86.44% at 0.1 mg/ml and 85.85% at 0.05 mg/ml.

In the DPPH assay illustrated in Fig. 2, the preliminary DPPH experiment demonstrated that only the *S. marcescens*-TSB extract had a moderate activity of 47.31% at 0.1 mg/ml. The *S. marcescens*-NB extract displayed a negligible DPPH scavenging activity. The results obtained here align with earlier studies, which reported that most crude extracts, including bacterial endophyte extracts, usually have low antioxidant activity against free radicals such as DPPH, until subsequent stages of purification or fractionation, which increases the concentration of their active principles [28-30].

### Changes in Biological Activity according to Cultivation Period of *S. marcescens* AI-N-1

Large-scale cultures of *S. marcescens* were then prepared and centrifuged to separate the supernatants and the cell pellets, which were extracted using organic solvents. Biological assays were carried out for the second time with the new extracts. During metabolite production, a colour change was noticed in the culture day after day. After one day of cultivation, the culture was pink to deep red. After two days of cultivation, the broth cultures transitioned from an intense pink to a lighter pink. After 7 days of culture, the broth containing *S. marcescens* ranged in colour from orange to dark mustard (Fig. S1). The fluctuation in culture colour, day after day, simply indicated that the endophytic bacterium *S. marcescens* produced various secondary metabolites, which is why the designations D7, D2, and D1 were assigned. *S. marcescens* D7 signified that the extract was produced after 7 days of culture. D2 signified that the extract was produced after 2 days of culture. D1 signified that the extract was produced after 1 day (24 h) of culture. Indeed, Heu *et al*. reported that Prodigiosin and other metabolites produced by *S. marcescens* are based on temperature-dependent and time-dependent factors [25].

As shown in Table 2, regarding the antibacterial susceptibility test, some extracts, following separation into supernatant and pellet fractions, exhibited activity levels statistically similar to those of the original *S. marcescens*-NB sample (*p* = 0.001). Notably, at 5 mg/ml, the *S. marcescens*-D7 pellet extract inhibited *Bacillus subtilis* by 78.45%, closely matching the 79.79% inhibition observed with the original *S. marcescens*-NB extract (*p* = 0.001). Similarly, against *S. typhi* at 5 mg/ml, the *S. marcescens*-D7 pellet extract inhibited *S. typhi* by 79.87%, which was higher than the 77.04% inhibition shown by the original *S. marcescens*-NB extract (*p* = 0.001). In fact, the *S. marcescens*-D7 pellet extract was obtained after 7 days of culture, just like the original *S. marcescens*-NB extract, which was not fractionated and was extracted as a whole culture after the same incubation period. This suggests that the antibacterial metabolites present in the original extract are likely also retained in the D7 pellet fraction. Relatively, among the supernatant samples, *S. marcescens*-D2-supernatant exhibited the highest activity at 5 mg/ml, inhibiting *B. subtilis* and *S. typhi* by 71.14% and 70.72%, respectively. These results indicate that antibacterial metabolites are present in both the pellet and the supernatant, with the pellet extract showing activity comparable to the whole extract. The consistency of inhibition across both Gram-positive (*B. subtilis*) and Gram-negative (*S. typhi*) pathogens highlights the broad-spectrum efficacy of *S. marcescens* metabolites. These results are supported by previous studies demonstrating that *S. marcescens* strains could produce various bioactive metabolites, including prodigiosin and serrawettin homologues, which have effectively suppressed the proliferation of both Gram-negative and Gram-positive pathogens [23, 31]. These findings further confirm the broad-spectrum efficacy of metabolites generated by *S. marcescens*, whether produced after one day, two days, or seven days of cultivation, which is why the establishment of their metabolite profiling was necessary.

**Fig. 3:**
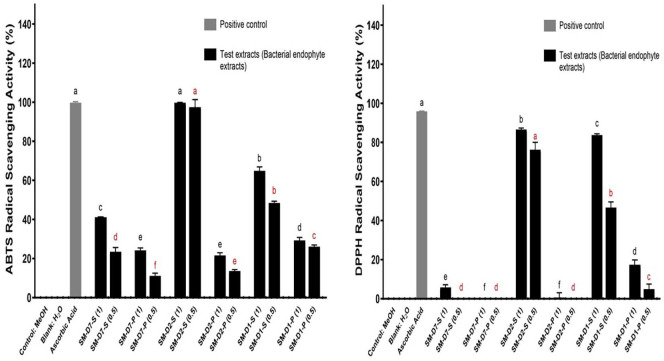
DPPH and ABTS radical scavenging activities of extracts according to cultivation periods of *S. marcescens* AI-N-1 Key: SM= *S. marcescens*; S= supernatant; P= pellet; D1= extract produced after 1 day (24 h) of culture; D2= extract produced after 2 days of culture; D7= extract produced after 7 days of culture; (1) = final concentration of 0.1 mg/ml; (0.5) = final concentration of 0.05 mg/ml. Different superscripts (^a–f^) indicate significant differences based on Duncan’s test at *P* ≤ 0.05. Superscript colors indicate separate statistical analyses: black (0.1 mg/ml) and orange (0.05 mg/ml). Units are expressed consistently as mg/ml. Positive control (1 mM ascorbic acid, ~0.176 mg/ml) is presented for comparison.

As illustrated in Fig. 3, the tested endophytic extracts, particularly upon fractionation into supernatant and pellet extracts, demonstrated striking activity in the case of the antioxidant studies. For example, *S. marcescens*-D2-supernatant extract demonstrated an ABTS scavenging activity of 99.64% at 0.1 mg/ml and 97.47% at 0.05 mg/ml, which were higher than the initial *S. marcescens* sample (*p* < 0.001). On the other hand, *S. marcescens*-D1-supernatant extract exhibited 64.85% scavenging activity at 0.1 mg/ml and 48.39% at 0.05 mg/ml.

Concerning the DPPH assay shown in Fig. 3, *S. marcescens*-D2-supernatant extract was found to give a DPPH scavenging activity of 86.61% at 0.1 mg/ml and 76.21% at 0.05 mg/ml. Similarly, *S. marcescens*-D1-supernatant extract exhibited 83.63% scavenging at 0.1 mg/ml and 46.61% at 0.05 mg/ml. These findings reflect a quite reasonable antioxidant potential compared with 1 mM Ascorbic acid (~0.176 mg/ml), which displayed a DPPH scavenging activity of 94.75% (*p* < 0.001).

Recent studies have investigated the antioxidant capabilities of metabolites from *S. marcescens*. Arivizhivendhan *et al*. demonstrated that prodigiosin produced by *S. marcescens* entirely neutralised DPPH and ABTS radicals at 10 mg/l [32]. Ibrahim *et al*. also reported strong antioxidant activity of prodigiosin, showing 97.2% DPPH radical scavenging at a concentration of 400 μg/ml after 120 min [33]. Similarly, Arivo *et al*., who, while investigating endophytic *Bacillus tequilensis* extracts isolated from *Leea indica* leaves, after fractionation, obtained high antioxidant activity with IC_50_ = 59.02 μg/ml and emphasised the role of flavonoids and phenolics in the endophytic extracts as being important for free radical scavenging [34]. Therefore, the purification and fractionation of extracts are critical elements to consider when assessing the antioxidant activity of endophytic extracts [35].

These results underscore the potential of *S. marcescens* metabolites as natural antioxidants with prospective uses in health promotion, disease prevention, and food preservation. Therefore, the metabolite profiling of the *S. marcescens* samples becomes necessary.

### Chemical Profiling and Identification of Secondary Metabolites according to Cultivation Period of *S. marcescens* AI-N-1

High-resolution mass spectrometry coupled with tandem mass spectrometry (HR-MS/MS) was employed for metabolite profiling to identify bioactive compounds in the bacterial endophyte *S. marcescens* extracts towards exploiting and understanding their biological activity. The *S. marcescens*, an endophytic bacterium isolated from *A. indica*, was found to produce various compounds.

The antibacterial activity of extracts from both the pellet and supernatant varied depending on the cultivation period. The pellet extracts exhibited the highest antibacterial activity at the early stage of cultivation but maintained notable activity even after 7 days. In contrast, the supernatant extracts showed the strongest antibacterial effect on day 2 of cultivation.

**Fig. 4:**
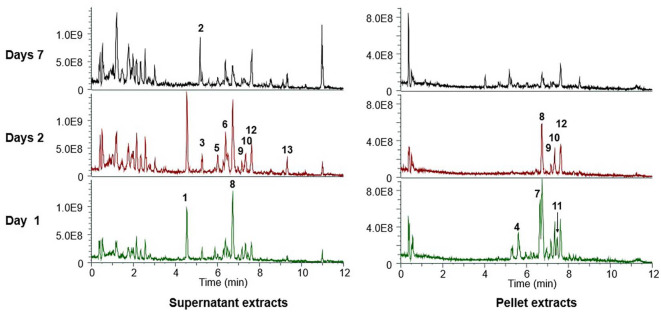
LC-MS chromatograms of culture extract from *S. marcescens* AI-N-1. Extracts were obtained from both supernatant (left) and pellet (right) after 1 day, 2 days, and 7 days of cultivation. The numbered peaks correspond to the compounds listed in Table 3.

As shown in the LC-MS results (Fig. 4 and Table 3), dynamic changes in metabolite production occurred throughout the cultivation period. Specifically, serranticin (**1**) was detected in the early-stage supernatant but completely disappeared by day 7. The characteristic red pigment prodigiosin (**4**), a representative secondary metabolite of *Serratia* species, was only detected in the pellet on day 1. In contrast, the cyclic lipopeptides known as serratamolides, including serratamolide A (**8**), were actively produced in the early phase but were consistently detected in both the pellet and supernatant throughout the entire cultivation period.

These observations suggest that the antibacterial effects of the extracts at different time points are likely attributable to a complex interplay of multiple bioactive compounds whose production varies throughout the cultivation period. These correlations suggest, but do not confirm, a causal relationship between metabolite changes and bioactivity. Alternative explanations, such as matrix effects, concentration shifts, medium differences, or metabolite synergism, may also account for the observed variations.

This variation in metabolites aligns with observations from metabolomic studies on *S. marcescens* strains, which indicated that variations in culture conditions and timeframes activate cryptic biosynthesis-related gene clusters, leading to diverse metabolite profiles [36].

Additionally, the HR-MS/MS analysis revealed the potential production of novel serratamolide derivatives (putative new serratamolides). The novel serratamolide-like peaks are considered tentative (MSI Level 2) annotations, supported by library matches without co-injected standards. Two compounds detected at retention times of 6.05 min and 7.66 min exhibited [M+H]^+^ ions at *m/z* 533.3433 and 571.3974, respectively, corresponding to the molecular formulas C_26_H_48_O_9_N_2_ and C_30_H_54_O_8_N_2_. Comparison of their MS/MS fragmentation patterns with that of serratamolide A revealed similar fragmentation features, suggesting that these two compounds are previously unreported serratamolide derivatives (Fig. S3).

The presence of prodigiosin, a tripyrrole alkaloid pigment, may help explain the remarkable antimicrobial activity shown by *S. marcescens* AI-N-1, particularly the activity observed in the pellet extracts during the early stage of cultivation (Table 2). This is because alkaloids are secondary metabolites that are nitrogen-containing base molecules that can be produced by endophytes with a broad range of bioactivities, including antimicrobial effects, owing to their chemical and structural varieties [37]. Prodigiosin is a well-documented major metabolite produced by Serratia strains, recognised for its broad-spectrum bioactivities, including antibacterial, antifungal, antioxidant, and anticancer properties. Recent studies have highlighted the potential of *S. marcescens* as a source of bioactive secondary metabolites with diverse applications. Baráti-Deák *et al*. highlighted the potent antimicrobial activity of prodigiosin in their study, further corroborating its bioactive importance [38]. Additionally, Nguyen *et al*. reported the strong anticancer activity of prodigiosin, demonstrating an average IC_50_ value of 2.1 μg/ml across 60 cancer cell lines, highlighting its potency for disrupting cellular processes such as apoptosis induction [39].

Serratamolides and their derivative compounds are lipopeptides that have already been documented for their excellent antimicrobial activity [40]. This could also be responsible for the impressive antimicrobial activity observed (Tables 1 and 2). In comparison with previous studies, advanced metabolomics techniques identified serratamolides, glucosamine derivatives, prodigiosin, and serratiochelin A in both pigmented and non-pigmented strains [36]. These metabolites exhibited broad-spectrum antimicrobial activity against multidrug-resistant pathogens, with prodigiosin showing the highest potency [23].

### Online HPLC–ABTS+ Analysis

**Fig. 5:**
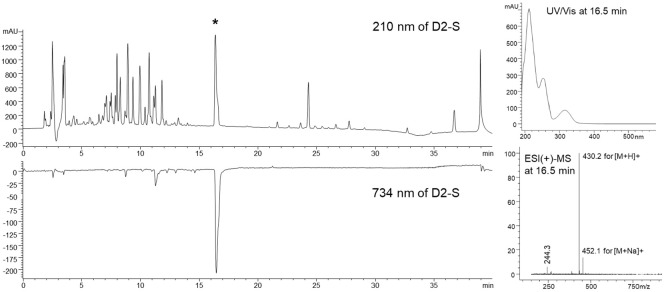
Online HPLC-ABTS+ chromatogram of culture extract from *S. marcescens* AI-N-1 after 2 days of cultivation. The upper chromatogram was recorded at 210 nm, while the lower chromatogram was monitored at 734 nm to detect ABTS radical scavenging activity. The UV/Vis absorption and ESI (+) mass spectra on the right correspond to the peak at 16. 5 min, indicated by an asterisk.

To identify the active antioxidant compound produced by *S. marcescens* AI-N-1, an online antioxidant HPLC analysis was performed. This technique allows for the rapid screening of radical-scavenging compounds without the need for prior purification, by simultaneously introducing the ABTS radical solution through an additional pump during the HPLC experiment. As illustrated in Fig. 5, the analysis revealed a strong negative peak at a retention time of 16.5 min on the detector set at 734 nm, indicative of ABTS radical scavenging activity. Based on the UV/Vis absorption spectrum and ESI mass spectrum, the compound corresponding to this retention time was identified as serranticin (**1**). The serranticin peak (RT = 16.5 min) displayed λmax at 210 and 280 nm with [M+H]+ = 430.1601, consistent with reported features for serranticin. This compound has already shown strong antioxidant and antitumor activities [41]. The detection of serranticin as the major antioxidant compound in *S. marcescens* AI-N-1 underscores the strain’s potential as a natural source of pharmacologically relevant secondary metabolites. These results not only validate the utility of the Online HPLC–ABTS^+^ approach for antioxidant screening but also suggest that *S. marcescens* AI-N-1 could serve as a promising candidate for further development in antioxidant-based therapeutics.

## Conclusion

Biological evaluation of bioactive compounds from endophytic bacteria isolated from Nigerian medicinal plants has not been fully explored. The present study demonstrated that the endophytic bacterium *S. marcescens*, isolated from *A. indica*, produced various bioactive compounds with significant antibacterial, biofilm inhibitory, and antioxidant activities. The crude extracts possessed broad-spectrum antibacterial activity against Gram-positive and Gram-negative bacteria, in addition to excellent inhibition of biofilm formation. Fractionation improved their antioxidant activity, especially in the DPPH test. HR-MS/MS analysis revealed the presence of prodigiosin, serranticin, serratamolides, and other metabolites, including new lipopeptides, supporting the broad-spectrum activity of the extracts. Isolation and structural elucidation of the novel compounds identified, along with *in vivo* experiments to demonstrate their therapeutic relevance, should be the aim of future studies. In summary, our study highlights the significance of exploring plant-associated endophytes as sustainable sources of new pharmacologically active molecules.

## Figures and Tables

**Table 1:** Preliminary Antibacterial Susceptibility Test results of *S. marcescens* crude extracts.

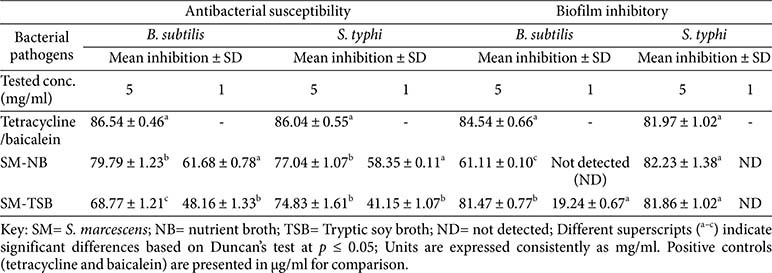

**Table 2:** Antibacterial activity of extracts according to cultivation periods of *S. marcescens* AI-N-1.

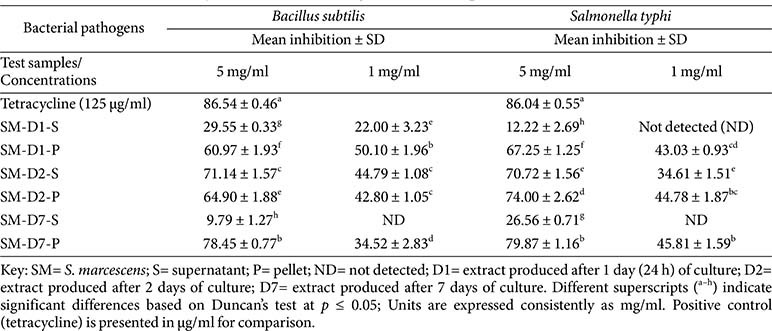

**Table 3:** Compounds identified from *S. marcescens* AI-N-1 using HR-MS/MS.